# p27^Kip1^ Is Required to Mediate a G1 Cell Cycle Arrest Downstream of ATM following Genotoxic Stress

**DOI:** 10.1371/journal.pone.0162806

**Published:** 2016-09-09

**Authors:** Erica K. Cassimere, Claire Mauvais, Catherine Denicourt

**Affiliations:** Department of Integrative Biology and Pharmacology, The University of Texas Health Science Center, Houston, Texas, United States of America; German Cancer Research Center, GERMANY

## Abstract

The DNA damage response (DDR) is a coordinated signaling network that ensures the maintenance of genome stability under DNA damaging stress. In response to DNA lesions, activation of the DDR leads to the establishment of cell cycle checkpoints that delay cell-cycle progression and allow repair of the defects. The tumor suppressor p27^Kip1^ is a cyclin-CDK inhibitor that plays an important role in regulating quiescence in a variety of tissues. Several studies have suggested that p27^Kip1^ also plays a role in the maintenance of genomic integrity. Here we demonstrate that p27^Kip1^ is essential for the establishment of a G1 checkpoint arrest after DNA damage. We also uncovered that ATM phosphorylates p27^Kip1^ on a previously uncharacterized residue (Ser-140), which leads to its stabilization after induction of DNA double-strand breaks. Inhibition of this stabilization by replacing endogenous p27^Kip1^ with a Ser-140 phospho-mutant (S140A) significantly sensitized cells to IR treatments. Our findings reveal a novel role for p27^Kip1^ in the DNA damage response pathway and suggest that part of its tumor suppressing functions relies in its ability to mediate a G1 arrest after the induction of DNA double strand breaks.

## Introduction

Cells in all organisms are constantly subjected to exogenous and endogenous sources of DNA damaging agents. The maintenance of genomic integrity is essential to preserve proper cellular function and prevent the transmission of DNA lesions, which contribute to aging and diseases such as cancer. To ward off threats posed by DNA damage, mammalian cells have evolved a complex signaling network, called the DNA-damage response (DDR), to sense the damage, delay cell cycle progression and repair the defects or induce programmed cell death if the lesions are too intensive [1]. The phosphatidylinositol 3-kinase-like kinase (PIKK) family, which includes ATM, ATR, and DNA-PK, plays central roles in sensing and responding to DNA insults [2]. ATM plays a critical role in initiating the cellular signaling cascade in response to DNA double strand breaks (DSB). Once activated, ATM phosphorylates a series of downstream substrates involved in the establishment of cell cycle checkpoints that ultimately leads to the inactivation of cyclin/cyclin-dependent kinase (CDK) complexes and consequently cell cycle arrest [3].

The G1 cell cycle checkpoint primarily prevents damaged DNA from being replicated. One of the most studied responses to DSB in G1 involves ATM and its direct substrate Chk2 promoting the stabilization of p53, which in turn induces the transcriptional activation of p21^Cip1^, a member of the CIP/KIP family of CDK inhibitor (CKI) [3–5]. p21^Cip1^ binds to and inhibits the activity of cyclin E/CDK2 complexes thereby arresting cells at the G1/S transition. In parallel, this delayed transcriptional response is accompanied by a rapid, but transient, ATM-, Chk2- and p38-dependent degradation of cyclin D and the Cdc25A phosphatase that removes the inhibitory phosphates on Cdk2 at T14 and Y15 [6–10].

p27^Kip1^ is also a member of the CIP/KIP family of CDK inhibitors [11]. However, unlike p21^Cip1^, p27^Kip1^ has been mostly studied for its roles in inhibiting G1 progression and maintaining cell quiescence in response to anti-proliferative signals or terminal differentiation [12–14] and reviewed in [11, 15]. p27^Kip1^ is not a p53 transcriptional target but rather, its functions are predominantly regulated postranslationally through phosphorylation events that affect protein stability, degradation and subcellular localization. Although p21^Cip1^ is though to be the main CKI induced by DNA damage, multiple studies have suggest that p27^Kip1^ also plays a crucial role in the maintenance of genomic stability. For example, p27^kip1^ null and heterozygous mice show increased susceptibility to tumor formation in multiple tissues when challenged with chemical carcinogens or γ-radiation [16]. In addition, compared with wildtype littermates, these mice also display higher rates of mutation induced by carcinogens and had an impaired G2/M arrest following low doses of ionizing radiation [17]. Though the molecular mechanisms by which p27^Kip1^ regulates genomic integrity are not very well understood, p38 MAPK-dependent stabilization of p27^Kip1^ was shown to be essential for a G2/M checkpoint arrest in response to prolonged exposure to DNA breaks [18].

In this study we demonstrate that p27^Kip1^ is essential for the establishment of the G1 cell cycle checkpoint arrest preventing cells from entering S-phase after DNA damage. We found that ATM kinase directly phosphorylates p27^Kip1^ at a previously uncharacterized residue (Ser-140) in a rapid and transient manner after the induction of DNA double-strand breaks. Phosphorylation at S140 increases p27^Kip1^ protein half-life, which is required to maintain cell survival after ionizing radiation treatments. Our data provide a novel aspect of the tumor suppressing functions for p27^Kip1^ where it directly participates in the G1 DNA damage signaling cascade downstream of ATM.

## Materials and Methods

### Cell Culture, tumorspheres isolation, and RNA interference

Cells (MCF7, MDA-MB-231, HFF and U2OS) were cultured at 37°C with 5% CO_2_ in Dulbecco's modified Eagle's medium (DMEM) high glucose (Hyclone) supplemented with 5% fetal bovine serum (FBS, Hyclone) and penicillin-streptomycin. For tumorspheres cultures, cells were seeded on Ultralow adhesion culture dishes (Corning) with mammosphere media (DMEM F12 (1:1), 10 ng/ml bFGF, 20 ng/ml EGF, 5 ug/ml insulin and 1x B-27 supplement (Invitrogen)). Synthetic short interfering RNA (siRNA) oligonucleotides (Sigma) were delivered into cells with Lipofectamine RNAi Max (Invitrogen). 10 nM of siRNA oligonucleotides were transfected using the reverse transfection protocol according to the manufacturer’s instructions. The following siRNA oligonucleotide targeting sequences were used: p27^Kip1^ (5’-GAAUGGACAUCCUGUAUAA-3’); p21 (5’-GAUGGAACUUCGACUUUGU-3’); ATM (5’-AACAUACUACUCAAAGACAUU-3’). For small hairpin RNA (shRNA), nontargeting scramble (5’-CCTAAGGTTAAGTCGCCCTCGCTCTAGCGAGGGCGACTTAAC CTTAAG-3’) and human p27^Kip1^ (5’-CCGGGCGCAAGTGGAATTTCGATTTCTCGAGAAATCGAAATTCCACTTGCGCTTTTTG-3’) sequences were cloned in pLKO.1-puro lentiviral vector. For virus production, HEK293T cells were transfected with pLKO.1-puro, pVSVG, pMDL, and pREV plasmids in a 3:1:1:1 ratio [19]. Medium-containing virus was collected 48 h and 72 h after transfection and used to infect cells for shRNA expression. For the p27^Kip1^ knockdown-rescue experiments, MDA-MB-231 cells were infected with scramble or p27^Kip1^ shRNA expressing lentiviruses and selected with puromycin for 4 days. Pools of resistant clones were subsequently infected with retroviruses expressing shRNA resistant Myc-p27WT or Myc-p27S140A (cloned in pBABE-Hygro) and selected with hygromycin for 4 days.

### Plasmids

The myc-p27WT (pCMV-myc vector; Clontech) construct has been previously described in [20]. All p27^Kip1^ mutant constructs were generated using the QuikChange II Mutagenesis kit (Stratagene). For the p27^Kip1^ knockdown-rescue experiments, the Myc-p27WT or Myc-p27S140A plasmids (in pCMV-myc vector) were made shRNA resistant by introducing silent mutation by PCR mutagenesis with the following primers: Forward (5’- gag gcg agc cag cgg aaa tgg aac ttt gac ttt cag aat cac - 3’) and Reverse (5’- gtg att ctg aaa gtc aaa gtt cca ttt ccg ctg gct cgc ctc - 3’). The Myc-p27WT and Myc-p27S140A shRNA-resistant were subsequently sub-cloned in pBABE-Hygro.

### Cell treatments

H_2_O_2_ (Hydrogen peroxide, 0–0.2 mM diluted fresh from a 30% solution stock; EMD Millipore), CHX (cycloheximide, 50 μg/ mL in DMSO; Calbiochem), KU-55933 (ATM kinase inhibitor, 10 μM in DMSO; EMD Millipore), IR treatment (Ionizing radiations, 0–10 Grays; gamma rays from a cesium source (JL Shepherd and Associates).

### Clonogenic cell survival and multicolor competition assays

The clonogenic cell survival assay was performed as described in [21]. Briefly, cells grown in monolayer or suspension spheres were subjected to 0, 4 or 6 Gy of IR. After 1 hour of recovery time, cells were trypsinized and seeded at different dilutions on adherent dishes. After 10–14 days, the plates were stained with Gentian Violet, air-dried and the colonies were counted to calculate the plating efficiency (number of colonies counted/number of cells plated). For the multicolor competition assay, RFP expressing cells were transfected with a control siRNA and GFP expressing cells with a p27-specific siRNA. Three days post transfection, GFP and RFP cells were mixed in a 1 to 1 ratio before treatment with IR. After 7 days in culture, cells were analyzed by flow cytometry using BDFortessa cell analyzer. The relative survival of the control siRNA-treated cells was set to 100%.

### Immunoprecipitation, immunoblotting and antibodies

Tris-HCl pH 7.6, 150mM NaCl, 1% NP-40, 1% Triton X-100, 1% sodium deoxycholate, and 0.1% SDS) plus protease inhibitors (aprotinin, leupeptin, AEBSF) on ice for 15 minutes. Lysates were cleared by centrifugation at 22 000 × g for 10 minutes at 4°C and the supernatant was incubated with 1 μg of antibody for 1 hour with rotation at 4°C. Protein G-sepharose beads (Invitrogen) were added for another 1 hour with rotation 4°C. An anti-myc 9E10 antibody (Santa Cruz Biotechnology) was used to immunoprecipitate myc-p27 WT and myc-p27 S140A. For immunoblotting, cells were lysed in RIPA buffer (25mM Tris-HCl pH 7.6, 150mM NaCl, 1% NP-40, 1% Triton X-100, 1% sodium deoxycholate, and 0.1% SDS) plus protease inhibitors (aprotinin, leupeptin, AEBSF) and phosphatase inhibitor cocktail (ThermoFisher) for 15 minutes on ice. Lysates were cleared by centrifugation at 22 000 × g for 10 minutes at 4°C. Protein concentrations were evaluated with the BCA kit (Pierce). Proteins were separated by SDS-polyacrylamide gel electrophoresis (SDS-PAGE) and transferred to a nitrocellulose membrane (Bio-Rad). The following antibodies were used: p27^Kip1^ (Sc-528, Santa Cruz Biotechnology), β-actin (Sc-69879, Santa Cruz Biotechnology), CDK2 (Sc-163, Santa Cruz Biotechnology), Chk2 (Cell Signaling), pThr68-Chk2 (Cell Signaling), ATM (D2E2, Cell Signaling), Myc (9E10, Santa Cruz Biotechnology), GAPDH (Sc-47724, Santa Cruz Biotechnology). The pSer140-p27^Kip1^ rabbit antibody was generated by Cell Signaling Technology, Inc., Danvers, Massachusetts. Densitometry analyses were performed using ImageJ version 1.46r from the National Institutes of Health.

### Kinase assays

For in vitro phosphorylation assessment of p27^Kip1^ at S140, recombinant GST-p27WT and GST-p27S140A purified from E. coli BL21 were used as substrates and incubated with purified DNA-dependent protein kinase (DNA-PK) (Promega). The kinase reactions were performed as followed: 100 ng of substrate was incubated with 10 units of DNA-PK in a 50 ul reaction mixture containing 50mM HEPES (pH 7.5), 100 mM KCl, 10 mM MgCl_2_, 0.2 mM EGTA, 0.1 mM EDTA, 1 mM DTT, 0.2 mM ATP, 2 uCi [γ-^32^P]ATP (3 000Ci/mmol), 80 ug/ml BSA and with or without 10 ug/ml calf thymus DNA. Samples were incubated at 30°C for 10 minutes and the reactions were stopped by adding 20 ul of 30% acetic acid. For the CDK2 kinase assays, cells transfected with control or p27^Kip1^ siRNAs were lyzed in ELB (50 mM HEPES pH 8.0, 250 mM NaCl, 2 mM EDTA, 0.5% NP-40) plus protease inhibitors (aprotinin, leupeptin, AEBSF). Lysates were cleared by centrifugation at 22 000 × g for 10 minutes at 4°C and the supernatant was incubated with 1 μg of CDK2 antibody for 1 hour with rotation at 4°C. Protein G-sepharose beads (Invitrogen) were added for another 1 hour with rotation 4°C. Immunoprecipitates were washed 3 times in ELB and 2 times in kinase buffer (50 mM HEPES pH 8.0, 10 mM MgCl_2_) before being subjected to kinase reaction. Kinase activity was measured using histone H1 as a substrate in a reaction mixture containing 2 ug of Histone H1, 5 uCi of [γ-^32^P]ATP (3 000Ci/mmol), 50 uM ATP in kinase buffer that was incubated for 30 minutes at room temperature. For both the DNA-PK and CDK2 kinase assays, the reactions were separated by SDS-polyacrylamide gel electrophoresis (SDS-PAGE). The gel was stained with Coomassie blue before drying and exposure to film for autoradiography analysis.

### Cell synchronization and flow cytometry analysis

For synchronization in G0, cells were washed 3 times in phosphate buffered saline (PBS) and serum deprived for 72 hours. To synchronize cells in G1, serum deprived cells were re-stimulated by adding culture media supplemented with 5% FBS for 6 hours. Synchronization at the G1/S transition by double thymidine block was performed by treating the cells with 2 mM thymidine for 16 hours. After the first block, the thymidine was washed out and replaced with fresh culture media for 8 hours. Thymidine (2 mM) was added back for 16 hours for a second block. To obtain a G2/M population, cells were treated with 100 ng/ml of nocodazole for 16 hours. Cell cycle progression was assayed by determination of DNA content using propidium iodide (PI) and bromodeoxyuridine (BrdU) labeling followed by flow cytometry analysis. BrdU was added for 30 min, and cells were harvested and fixed with 70% ethanol overnight. Cells were centrifuged at 10,000 x *g* for 1 min and washed in BrdU wash solution (0.5% Tween 20, 0.5% bovine serum albumin (BSA) in PBS). Cells were resuspended in 2 N HCl for 20 min at room temperature, neutralized with 0.1 M sodium borate, and washed twice with BrdU wash solution. Cells were incubated with anti-BrdU antibody (BD 347580 mouse IgG) at room temperature for 30 min. Cells were washed 3 times in BrdU wash solution and incubated with Alexa Fluor 488 goat anti-mouse IgG (Invitrogen) for 30 min at room temperature. Cells were washed 3 times in BrdU wash solution and then treated with 100 ug/ml RNase and 25 ug/ml propidium iodide. Analyses were performed with a BD LSRFortessa instrument.

### Immunofluorescence microscopy

Cells grown on cover glass were fixed with 4% paraformaldehyde at room temperature for 15 minutes. Cells were then permeabilized with 0.1% Triton for 10 minutes at room temperature. All samples were blocked with 1% BSA for 30 minutes, washed with PBS, and incubated with the appropriate primary antibody for 1 hour. Cells were washed with PBS containing 0.05% Tween-20 and incubated with secondary antibody for 1 hour (Alexa-Fluor 488 goat anti-rabbit antibody, Invitrogen). Cells were washed again with PBS containing 0.05% Tween-20, stained with DAPI (Molecular Probes), and mounted on slides with Vectashield (Vector Labs). Fluorescence microscopy was performed on a Zeiss Axioskop 40 fluorescence microscope with a Plan-APOCHROMAT 63x/1.4 NA oil DIC objective. Images were acquired with an Axiocam MRm camera using the Axiovision Release 4.6 software. All microscopy was performed at room temperature, and all images were prepared in Adobe Photoshop and Adobe Illustrator.

## Results

### p27^Kip1^ is required to establish a G1 cell cycle arrest after ionizing radiations

**Fig 1:**
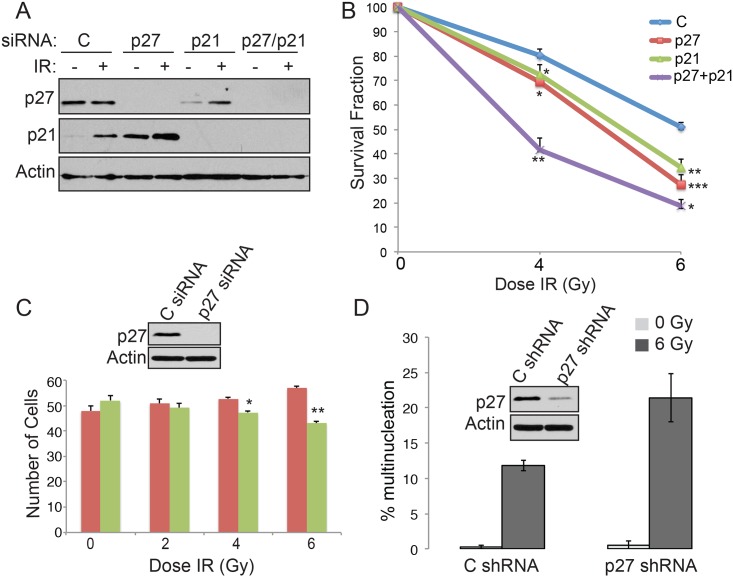
p27^Kip1^ is required for survival after DNA damage. (A) MCF7 cells were transfected with control (C), p27, p21 or p27 + p21 specific siRNAs. After 48h, the cells were subjected to 0, 4 or 6 Gy of IR. Knockdowns for the cells subjected to 0 (-) or 6 Gy (+) of IR were monitored by Western blotting analysis 24h post-irradiation with the indicated antibodies (left). (B) MCF7 cells treated with control (C), p27, p21 or p27 + p21 siRNAs and subjected to 0, 4 or 6 Gy of IR were seeded at low density for a clonogenic cell survival assay. The number of colonies was counted 11 days post IR treatment. The survival fraction was calculated relative to the plating efficiency of untreated cells. The data is presented as mean of 3 independent experiments ± SEM. Differences between groups were evaluated using two-tailed Student *t* tests among replicate experiments; *P<0.0197, **P<0.0035 and ***P = 0.0007. Single p27 or p21 knockdowns were each compared with control siRNA and double p27/p21 knockdowns were compared with single p27 knockdown for statistical analyses. (C) U2OS cells stably expressing RFP or GFP were transfected with control C or p27 specific siRNA, respectively. RFP and GFP cells were mixed together at a 1 to 1 ratio and subjected to 0, 2, 4 or 6 Gy of IR. After 7 days of culture, cells were collected and analyzed by flow cytometry to determine the cell number in each population. The data is presented as mean of 3 independent experiments ± SEM. Differences between groups were evaluated using two-tailed Student *t* tests among replicate experiments; *P = 0.0156 and **P = 0.0033. (D) U2OS cells expressing control C or p27 specific shRNA were subjected to 0 or 6 Gy or IR. Cells were fixed and stained with DAPI to assess nuclear morphology by fluorescence microscopy 7 days post-IR. Data is presented as mean of 3 independent experiments ± SEM.

Because p27^Kip1^ is essential to maintain genomic integrity and is a major regulator of Cdk2 activity at the G1/S transition, we hypothesized that p27^Kip1^ could be essential for the establishment of the G1 checkpoint that prevents cell from entering S-phase after induction of DNA double-strand breaks. To test this, we first assessed whether depletion of p27^Kip1^ affected cell viability by performing a clonogenic survival assay after gamma irradiation (IR) treatment. MCF7 cells transfected with p27^Kip1^ siRNA (Fig 1A) were markedly more sensitive to increasing doses of IR compared to control siRNA-treated cells (Fig 1B). This sensitivity to IR was comparable to the sensitivity observed with RNAi depletion of the CDK inhibitor p21, a p53 target gene with a well-characterized role in DNA damage checkpoint arrest (Fig 1A and 1B) [3]. Importantly, a double knockdown of p27^Kip1^ and p21 (Fig 1A) had a synergistic effect on cell sensitization to IR (Fig 1B), suggesting that p27^Kip1^ and p21 act in 2 distinct pathways activated in response to DNA damage. Using a multicolor competition assay [22] in U2OS cells, we also observed a similar sensitivity to DNA damage, where p27^Kip^ depleted cells (expressing GFP) showed a dose dependent decrease in survival relative to control siRNA transfected cells (expressing RFP) 7 days post-IR treatment (Fig 1C). Depletion of p27^Kip1^ also lead to an increase in cells with multi-nucleation 2 days after IR treatment (Fig 1D) suggesting the sensitivity to DNA damaging agents observed is due to genomic instability and that p27^Kip1^ is likely required to inhibit cells from replicating damaged DNA.

**Fig 2:**
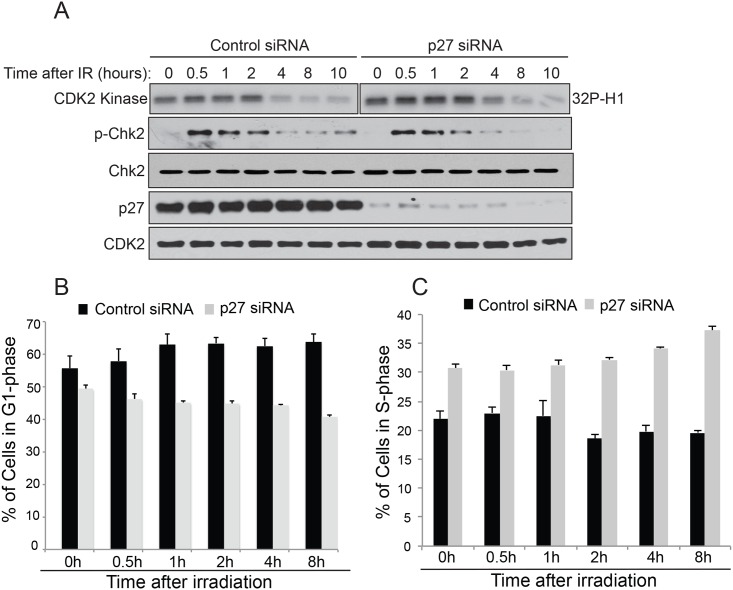
p27^Kip1^ is required to prevent S-phase entry after DNA damage. (A) Depletion of p27^Kip1^ leads to prolonged CDK2 activity after IR treatment. MCF7 cells were transfected with non-targeting control or p27 siRNAs for 72h before treatment with 6 Gy of IR. Cells were harvested at the indicated time points post IR and divided in 2. The first half was used to measure CDK2 kinase activity *in vitro* after immunoprecipitation using histone H1 as a substrate. The kinase assay reactions for the Control and the p27 siRNA samples were ran on 2 different gels but exposed on the same film for the same time duration. The second half of the RNAi transfected cells was used for Western blotting analysis with the indicated antibodies (left). (B) Cell depleted of p27^Kip1^ do not arrest in G1 and progress into S-phase after IR treatment. MCF7 cells were transfected with non-targeting control or p27 siRNAs for 72h before treatment with 0 or 6 Gy of IR. Cell were labeled with BrdU at the indicated time points and then fixed and stained with propidium iodide (PI). The percentage of cells in G1 at the indicated time points was measured by flow cytometry analysis of PI incorporation and (C). The percentage of cells in S-phase at the indicated time points was measured by flow cytometry analysis of BrdU incorporation. The data is presented as mean of 3 independent experiments ± SEM.

**Fig 3:**
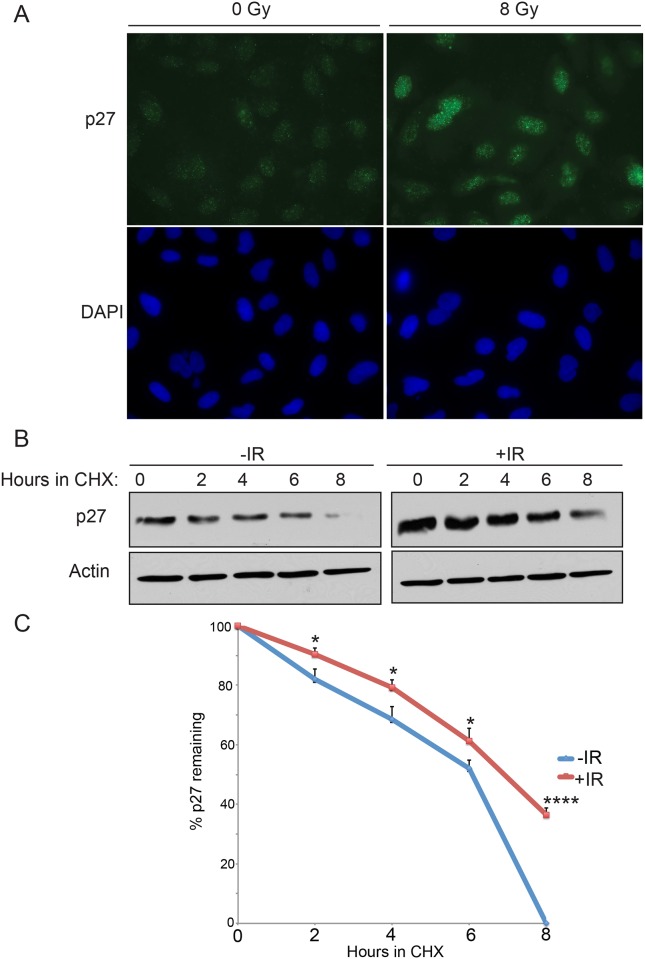
Induction of DNA damage by gamma-radiations increases p27^kip1^ protein stability. (A) MCF7 cells were subjected to 0 or 8 Gy or IR. 1h later, cells were fixed and analyzed by immuno-fluorescence microscopy with a p27 antibody and DAPI. (B) MCF7 cells were subjected to 0 (-IR) or 8 Gy (+IR) of IR in the presence of cycloheximide (CHX). Cells harvested at the indicated time points were analyzed by Western blotting with anti-p27 or Actin antibodies. (C) Densitometric quantification of the p27 levels normalized to actin from the Western blot in (B). The data is presented as mean of 3 independent experiments ± SEM. Differences between groups were evaluated using two-tailed Student *t* tests among replicate experiments; *P < 0.0352; ****P < 0,0001.

To test the hypothesis that p27^Kip1^ is essential to prevent S-phase entry after DNA damage, we depleted cells of p27^Kip1^ by RNAi and monitored CDK2 activity and cell cycle progression at different time points after IR treatment in MCF7 cells. Although CDK2 activity was slightly higher in p27^Kip1^ depleted cells at time 0h compared with control cells, the activity remained higher for up to 4h after irradiation in absence of p27^Kip1^ (Fig 2A and S1 Fig for densitometric quantification). Consistent with this observation, cells with depleted levels of p27^Kip1^ did not accumulate in G1 (Fig 2B) and continued to progress into S-phase after IR treatment (Fig 2C). Because p27^Kip1^ levels are high during G0 and fall gradually as cells enter G1 and transit in to S-phase [23], we asked whether DNA damage would lead to an increase in p27^Kip1^ protein stability to block progression into S-phase. As shown in Fig 3, we found this to be the case. As soon as 15 minutes after exposure to 8 Gy of IR we observed higher levels of nuclear p27^Kip1^ accumulating in treated cells (Fig 3A). Consistent with this, IR treatments increased p27^Kip1^ protein half-life within 2 hours after exposure (Fig 3B and 3C). Together, our data suggests that DNA damage signaling pathway stabilizes p27^Kip1^ to mediate the early establishment of a G1 DNA damage checkpoint.

### ATM phosphorylates p27^Kip1^ at Ser-140 after DNA damage

**Fig 4:**
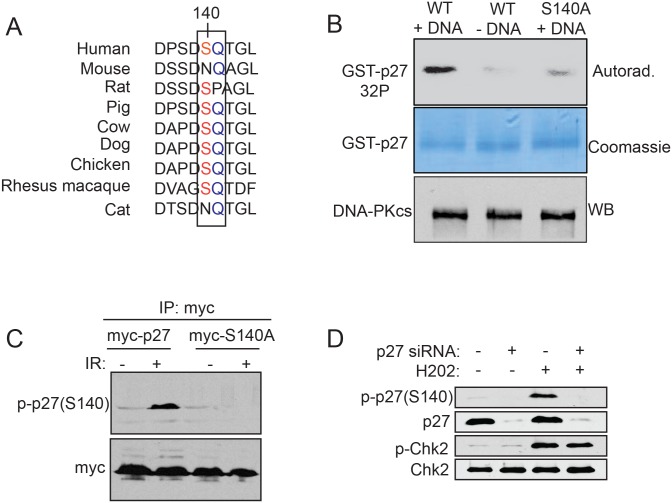
p27^Kip1^ is phosphorylated at Ser140 after induction of double-strand breaks. (A) Sequence alignment of human p27^Kip1^ (residue 136 to 144) across 8 model organisms. The ATM/ATR/DNA-PK SQ phosphorylation consensus is boxed. (B) p27^Kip1^ *in vitro* phosphorylation assay. GST-p27WT or S140A was incubated with purified DNA-PK in the presence of [γ-^32^P]ATP. Calf thymus DNA was added to activate the kinase where indicated (+DNA). A portion of the reaction was separated on SDS-PAGE and analyzed by autoradiography and another portion was separated on SDS-PAGE and analyzed by Western blotting with a DNA-PK antibody. (C) Specificity assessment of the p-p27(S140) antibody. MCF7 cells were transfected with either, empty vector (myc), myc-p27 or myc-p27S140A expressing constructs and subjected to 0 (-) or 6 Gy (+) of IR. 1 hour post IR, ectopically expressed p27^Kip1^ was immunoprecipitated with an anti-myc antibody and phosphorylation of p27^Kip1^ at S140 was assessed by Western blotting using a p27 S140 phospho-specific antibody (p-p27(S140)) as indicated on the left of the panel. (D) MCF7 cells were transfected with non-targeting control (-) or p27 siRNAs (+) for 72h before treatment with H_2_O_2_ (0.2 mM) for 1h to induce DNA double-strand breaks. Cells were processed for Western blotting analysis with specific antibodies as indicated on the left of each panel. All the panels from this figure are representative of 3 independent experiments.

Because we observed that the levels of p27^Kip1^ are rapidly stabilized in response to IR (Fig 3) we sought to determine whether p27^Kip1^ could actively participate in the DNA damage response pathway. Ataxia-telangiectasia mutated (ATM), DNA-dependent protein kinase catalytic subunit (DNA-PKcs), and ataxia-telangiectasia and Rad3-related (ATR) are members of the phosphoinositide-3-kinase-related protein kinase (PIKK) family that become immediately activated in response to DNA damage [2]. To determine if p27^Kip1^ could be a direct substrate for the members of that family of protein kinases in response to DNA damage, we queried publicly available phospho-proteome databases to identify p27^Kip1^ residues phosphorylated at the conserved ATM/ATR/DNA-PK phosphorylation consensus sites (ST/Q). Although human p27^Kip1^ contains 4 putative ATM/ATR/DNA-PK phosphorylation consensus sites (S56, S106, S140 and T162) only one of them (S140) has been found phosphorylated in a large-scale analysis of proteins phosphorylated in cells after IR treatment [24]. Surprisingly, this ATM/ATR/DNA-PK consensus site (S/TQ) is conserved in most vertebrate except for mouse, rat and cat (Fig 4A). To validate p27^Kip1^ S140 as a *bona fide* target for the ATM/ATR/DNA-PK kinases we performed an *in vitro* kinase assay using purified DNA-PK and either, recombinant GST-p27WT or the phospho-mutant GST-p27S140A as substrates. p27WT became phosphorylated only when DNA-PK was activated by the presence of double-stranded DNA (Fig 4B). Strikingly, this phosphorylation was lost to background level when p27S140A was used as substrate indicating that this serine residue is the main site recognized by the kinase, at least in this assay (Fig 4B). We did not observe any phosphorylation on GST alone (not shown).

To further investigate the role of S140 phosphorylation in the DNA damage response pathway, we used a newly developed S140 phospho-specific antibody to monitor phosphorylation of endogenous p27^Kip1^. We tested the specificity of the antibody in cells expressing myc-p27WT vs. myc-p27S140A or in cells transfected with a p27^Kip1^ siRNA. By Western blotting, the antibody showed positive reactivity at the expected size only on immuno-precipitated myc-p27WT (Fig 4C) or endogenous p27^Kip1^ (Fig 4D) and only from cells subjected to DNA damaging agents causing double-strand breaks (IR and hydrogen peroxide (H_2_O_2_)). The band was absent when immuno-precipitated mutant myc-p27S140A (Fig 4C) was used or when p27^Kip1^ levels were depleted by RNAi (Fig 4D), confirming the specificity of this antibody to phosphorylated S140 only. In addition, this antibody detected phosphorylated p27^Kip1^ at S140 in multiple cell lines, including primary fibroblasts (S2A Fig).

**Fig 5:**
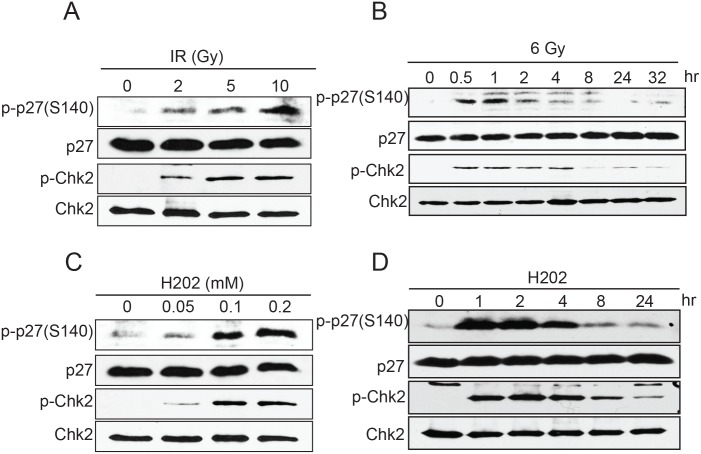
Dose and time-dependent phosphorylation of p27^Kip1^ at S140 by genotoxic agents. (A) MCF7 cells were subjected to different doses of ionizing radiations (IR). Cells were harvested 1h later and analyzed for p27^Kip1^ phosphorylation at S140 by Western blotting. (B) MCF7 cells were subjected to 6 Gy of ionizing radiations and harvested at the indicated time points. p27^Kip1^ phosphorylation at S140 was analyzed by Western blotting. (C) MCF7 cells were subjected to different doses of hydrogen peroxide (H_2_O_2_). Cells were harvested 1h later and analyzed for p27^Kip1^ phosphorylation at S140 by Western blotting. (D) MCF7 cells were treated with 0.2 mM of hydrogen peroxide (H_2_O_2_) for 1h. Cells were harvested and analyzed for p27^Kip1^ phosphorylation at S140 by Western blotting. For all panels the antibody used are indicated on the left and a phospho-Chk2 (Thr68) antibody was used to confirm the activation of the DNA damage response pathway. All the panels from this figure are representative of 3 independent experiments.

We next tested whether phosphorylation at S140 could be induced by different cellular stresses. Of all the conditions tested which included, glucose starvation, serum starvation, amino acid starvation, hypoxia, and heat shock, only DNA damage caused by etoposide (not shown), IR (Fig 5A and 5B) or H_2_O_2_ (Fig 5C and 5D) induced S140 phosphorylation in a dose-dependent manner. Remarkably, phosphorylation at S140 was not observed after UV treatment (not shown) or hydroxyurea-induced replication fork stalling (not shown) indicating that this phosphorylation appears to be specifically promoted by DNA double-strand breaks. The observed phosphorylation at S140 was rapid and transient appearing within 30 minutes of DNA damage induction and rapidly declining after 2–4 hours (Fig 5B and 5D). Moreover, immunofluorescence microscopy experiment revealed that S140 phosphorylated p27^Kip1^ localizes to the nucleus of cells as soon as 15 minutes after induction of DNA damage (S2B Fig). This kinetic of phosphorylation strongly suggests a role for p27^Kip1^ in the early stage of the DNA damage response pathway.

**Fig 6:**
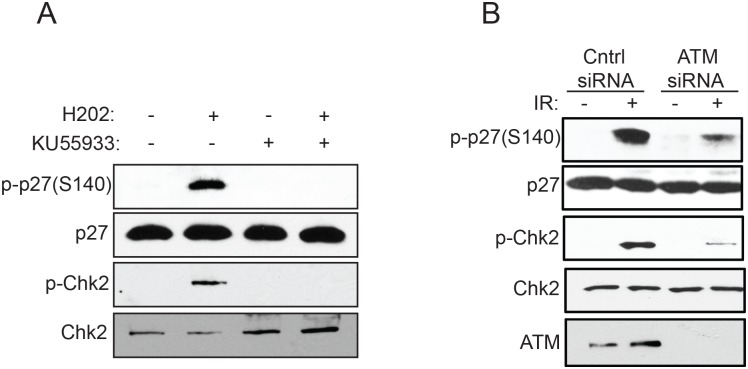
Phosphorylation of p27^Kip1^ at S140 is ATM-dependent. (A) Inhibition of ATM activity impairs phosphorylation of p27^Kip1^ at S140. MCF7 cells were incubated with DMSO (vehicle) or 10 μM of the ATM inhibitor KU55933 for 1h prior to treatment with H_2_O_2_ (0.2 mM). Cells were harvested 1h later and analyzed by Western blotting with the indicated antibodies (left). (B) RNAi depletion of ATM reduces phosphorylation of p27^Kip1^ at S140. MCF7 cells were transfected with non-targeting control (-) or ATM siRNAs (+) for 72h before treatment with 6 Gy of IR. Cells were harvested 1h later and analyzed by Western blotting with the indicated antibodies (left). A phospho-Chk2 (Thr68) antibody was used to confirm the activation of the DNA damage response pathway. All the panels from this figure are representative of 3 independent experiments.

ATM and DNA-PK are mainly activated in response to DNA double-strand breaks, whereas ATR activity is stimulated by stalled DNA replication forks and single-stranded DNA [1]. Our observation that phosphorylation of p27^Kip1^ at S140 is specifically induced by DNA double-strand breaks suggests that ATM or DNA-PK kinases, rather than ATR, are involved in phosphorylating p27^Kip1^ at S140. To test this we use the ATP-competitive inhibitor KU-55933, which is selective only for ATM and not ATR, and neither DNA-PK [25]. Pre-incubation of cells with KU-55933 for 1 hour prior to induction of DNA double-strand breaks with H_2_O_2_, completely abolished phosphorylation at S140 (Fig 6A). Knock down of ATM with specific siRNA also dramatically reduced the phosphorylation of p27^Kip1^ at S140 normally observed after IR treatment (Fig 6B), confirming that ATM is likely the main kinase responsible for phosphorylating p27^Kip1^ at this site.

### Phosphorylation at Ser-140 is required to maintain p27^Kip1^ protein stability after induction of DNA damage

**Fig 7:**
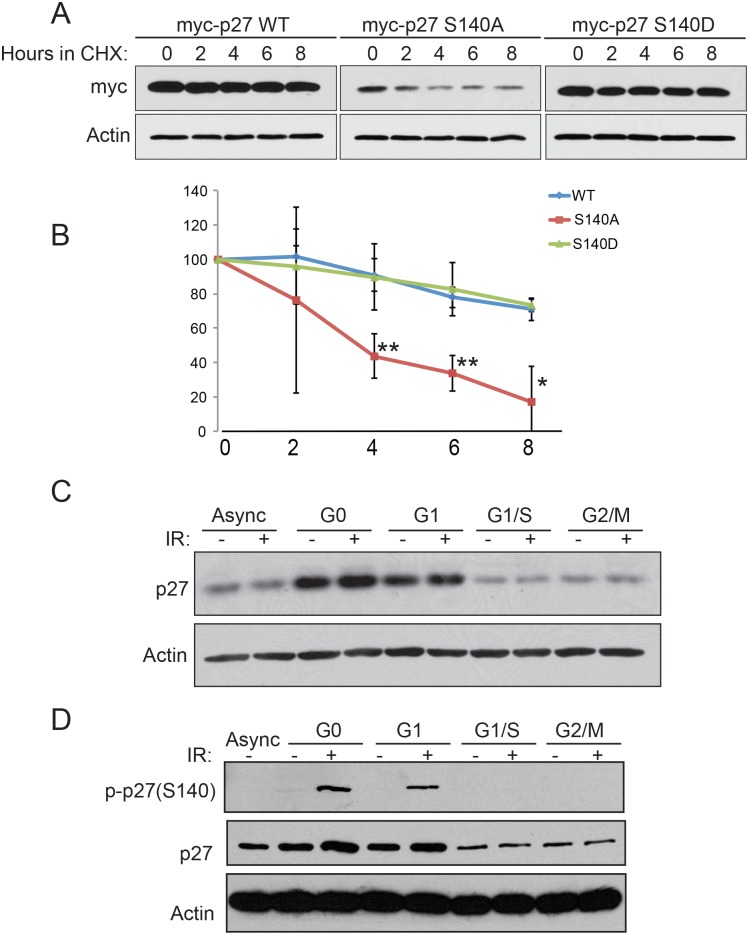
Phosphorylation of p27^Kip1^ at S140 regulates its protein stability in the G1-phase of the cell cycle. (A) Ectopically expressed p27^Kip1^ S140A mutant has a decreased half-life compared with the WT or phosphomimetic (S140D) versions. MCF7 were transfected with myc-p27WT, myc-p27S140A or myc-p27S140D expressing constructs for 24 hours and subjected to 6 Gy of IR. Cells were then treated with cycloheximide (CHX) for the indicated time after IR treatment. Each time points were analyzed by Western blotting with the indicated antibodies. (B) Densitometric quantification of the levels of myc-p27 from (A) normalized to actin. The data is presented as mean of 3 independent experiments ± SEM. Differences between groups were evaluated using two-tailed Student *t* tests among replicate experiments; **P<0.0072; *P = 0.0129. (C) MCF7 cells were synchronized in G0 by serum starvation; in G1 by serum starvation and released for 6h by the addition of serum; in G1/S by a double thymidine block; and in G2/M by nocodazole treatment. Cells growing in normal condition were used for the asynchronous (Async) population. The synchronized cells were treated with 0 (-) or 6 Gy of IR (+) and analyzed 1h after for total p27^Kip1^ protein level by Western blotting. The antibodies used are indicated of the left side of each panel. (D) MCF7 cells synchronized in the G0, G1 G1/S or G2/M of the cell cycle as in (C) were subjected to 0 (-) or 6 Gy of IR (+). Western blotting analysis of p27^Kip1^ S140 phosphorylation was examined 1h after. The antibodies used are indicated of the left side of each panel. All the panels from this figure are representative of 3 independent experiments.

To determine the biological function of p27^Kip1^ phosphorylation after DNA damage, we investigated whether the phosphorylation status of S140 had an influence on p27^Kip1^ protein stability. MCF7 cells were transiently transfected with a myc-tagged version of p27WT, a phospho-mutant (p27S140A) or a phospho-mimetic (S140D). Cells were subjected to 6 Gy of IR in the presence of cycloheximide to inhibit protein synthesis and the remaining p27^Kip1^ protein levels were assessed at different time points. Compared to WT, the S140A mutant exhibited a marked decrease in stability (Fig 7A and 7B). The S140D phospho-mimic had a comparable stability compared with the WT indicating that the charge of the aspartic acid fulfills the function of a phosphorylated residue and is important in regulating p27^Kip1^ protein half-life in response to DNA damage (Fig 7A and 7B). Together, our data indicates that phosphorylation at S140 is required to maintain p27^Kip1^ protein stability after DNA damage.

We next assessed whether the S140 phosphorylation and stabilization of p27^Kip1^ levels after DNA damage was cell cycle-dependent. Cells were synchronized in either, the G0, G1, G1/S or G2/M phases of the cell cycle (S3B Fig) and subjected to IR before analysis of total p27^Kip1^ levels. We observed that IR increased p27^Kip1^ protein level in a modest but reproducible manner specifically in G0 and G1 synchronized cells (Fig 7C and S3A Fig for densitometric quantification). Stabilization of p27^Kip1^ was not observed in cells late G1/S or at the G2/M transition of the cell cycle (Fig 7C). Concordantly, IR-induced phosphorylation of p27^Kip1^ at S140 was only detected in G0 and G1 cells (Fig 7D). These findings are consistent with a role for p27^Kip1^ in the establishment of the G1 DNA damage checkpoint arrest.

**Fig 8:**
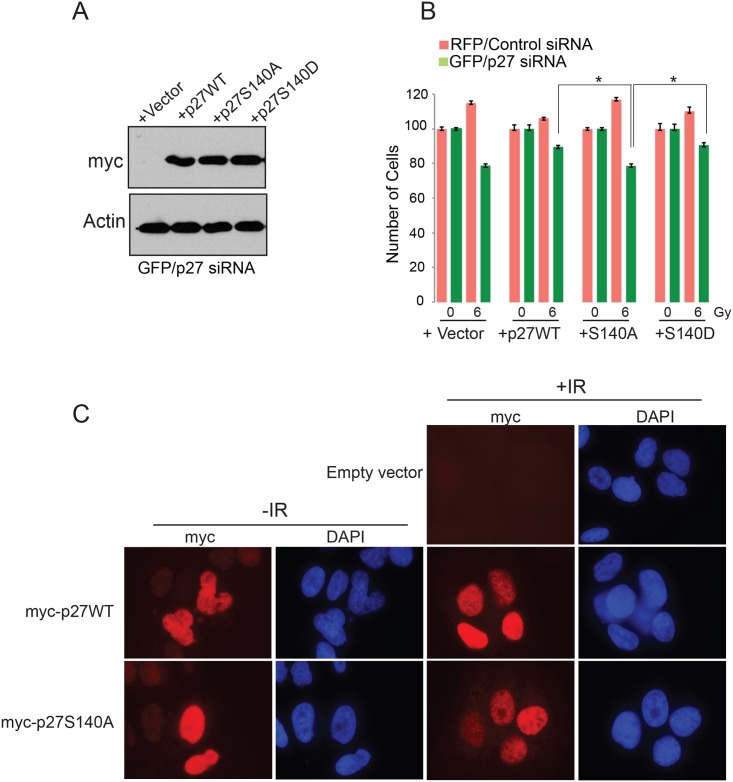
p27^Kip1^ S140 phosphorylation is required for survival after DNA damage. (A) U20S cells expressing RFP or GFP were transfected with control siRNA or a p27-specific siRNA, respectively. The next day the GFP/p27siRNA cells were transfected with vector alone, myc-p27WT, myc-p27S140A or myc-p27S140D and the RFP/control siRNA were transfected with vector alone only. Two days later the cells were harvested and a portion was analyzed by Western blotting to assess construct expression with the indicated antibodies (GFP cells only). (B) The rest of the cells were analyzed in a multicolor competition assay by flow cytometry. The relative survival of the control siRNA-treated cells was set to 100%. Data is presented as mean of 3 independent experiments ± SEM. Differences between groups were evaluated using two-tailed Student *t* tests among replicate experiments; *P = 0.0249. (C) U2OS cells were transfected with vector alone, myc-p27WT or myc-p27S140A expressing constructs. Two days later the cells were subjected to 0 (-IR) or 6 Gy of IR (+IR), fixed 1h later and the localization of p27WT and p27S140A was determined by immunofluorescence microscopy with a myc antibody and DAPI staining.

Our observation that depletion of p27^Kip1^ reduces cell survival after DNA damage suggests that its stabilization through phosphorylation at S140 is critical to mediate the G1 checkpoint arrest. To assess this we knockdown the levels of endogenous p27^Kip^ with siRNA in U2OS cells and replaced its expression with empty vector, myc-tagged p27WT, S140A or S140D mutants (Fig 8A). Using a similar multicolor competition assay as we used in Fig 1 we observed that, compared with cells expressing p27WT or the S140D mutant, cells expressing p27S140A displayed increased sensitivity to DNA damage as shown by their reduced number at day 7 post treatment with 6 Gy of IR (Fig 8B). The p27S140A mutant localized to the nucleus after IR (Fig 8C), confirming that the increased sensitivity to IR observed for the cells expressing the S140A mutant is likely due to a decrease of protein stability.

**Fig 9:**
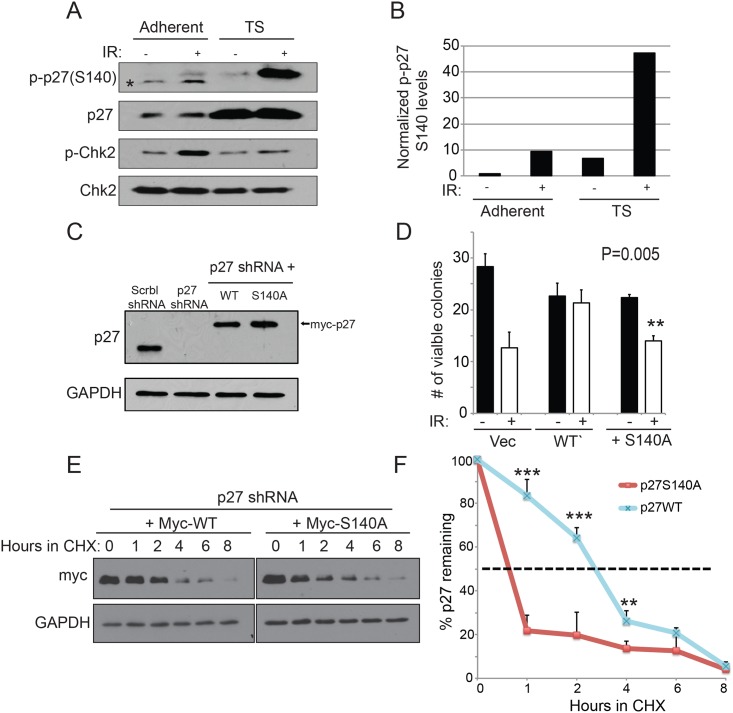
Stabilization of p27^Kip1^ through S140 phosphorylation is required for tumorsphere resistance to IR. (A) p27^Kip1^ is robustly phosphorylated at S140 in tumorspheres after IR treatment. MDA-MB-231 cells grown as adherent monolayer or isolated tumorspheres (TS) were subjected to 0 (-) or 6 Gy (+) of IR and analyzed 1 hour after by Western blotting with the indicated antibodies. The asterisk indicates an unspecific fragment detected by the p-p27(S140) antibody. (B) Densitometric quantification of the data in (A) where levels of p-p27(S140) were normalized to total p27 levels. (C) Western blotting analysis of the p27^Kip1^ knockdown rescue experiment. p27WT or S140A (myc-tagged) were re-introduced in MDA-MB-231 cells stably expressing p27^Kip1^ shRNA. A non-targeting scramble (Scrbl) shRNA was used as control. (D) MDA-MB-231 tumorspheres expressing p27S140A are more sensitive to IR. MDA-MB-231 tumorspheres expressing either, p27shRNA + vector alone, p27shRNA + p27WT or p27shRNA + p27S140A were subjected to 0 (-) or 6 Gy (+) of IR. After 1 hour of recovery time, the tumorspheres were trypsinized and replated on normal adhesion dishes. The number of colonies formed was counted 10 days post-IR. Data is presented as mean of 3 independent experiments ± SEM. Differences between groups were evaluated using two-tailed Student *t* tests among replicate experiments; **P = 0.005. (E) p27S140A has a decreased half-life compared to p27WT in tumorspheres subjected to IR. MDA-MB-231 tumorspheres expressing p27shRNA + p27WT or p27shRNA + p27S140A were subjected to 6 Gy of IR in the presence of cycloheximide (CHX). Cells harvested at the indicated time points were analyzed by Western blotting with anti-myc or GAPDH antibodies. (F) Densitometric quantification of the data in (E) where the levels of myc-p27 were normalized to GAPDH. The data is presented as mean of 3 independent experiments ± SEM. Differences between groups were evaluated using two-tailed Student *t* tests among replicate experiments; ***P < 0.0007; **P = 0,0058.

Breast cancer cells isolated as suspension tumorspheres have been shown to have high levels of p27^Kip1^ that was correlated with an increased survival to genotoxic agents [26]. We first verified that p27^Kip1^ was phosphorylated at this residue in tumorpsheres after DNA damage. Strikingly, we observed a much more robust phosphorylation at S140 in isolated tumorspheres compared with the parental culture grown as monolayer (Fig 9A and 9B). To test the role of S140 phosphorylation in mediating the increase in p27^Kip1^ stability required for survival after DNA damage in tumorspheres, we established stable MDA-MB-231 lines where endogenous p27^Kip1^ level depleted by shRNA was replaced by expressing either, wild-type (myc-p27WT) or phospho-mutant (myc-p27S140A) (Fig 9C). For both p27WT and p27S140A, several pools of stable clones that had similar levels of expression to endogenous p27^Kip1^ were combined together (Fig 9C). Tumorspheres expressing p27WT and p27S140A were exposed to 6 Gy of IR and after a recovery period the cells were passaged back in normal adhesion conditions to assess survival with a colony formation assay. Compared with tumorspheres expressing p27WT, tumorspheres expressing p27S140A were significantly more sensitive to IR (Fig 9D). Accordingly, the half-life of p27S140A was markedly decreased in IR-treated tumorspheres, specifically for the 1 and 2 hours time points (Fig 9E and 9F), which correlates with the fast and transient kinetics of S140 phosphorylation observed (Fig 5B and 5D). Together our findings demonstrate that the rapid and transient phosphorylation at S140 is important for p27^Kip1^ protein stabilization and that it contributes to cell survival after exposure to agent inducing DNA double-strand breaks.

## Discussion

We found that not only p27^Kip1^ is required for cell survival and the establishment of a G1 DNA damage checkpoint arrest, but that it also directly participate in the DNA damage response pathway downstream of ATM activation. Our data demonstrate for the first time that ATM phosphorylates p27^Kip1^ at S140, a previously uncharacterized residue, specifically after induction of DNA double strand breaks. Although S140 is not conserved in mouse or rat, it is possible that other ATM phosphorylation consensus sites located upstream (S106 and S121) serve the same function. We are currently investigating this possibility. Phosphorylation of p27^Kip1^ at S140 is essential to maintain p27^Kip1^ protein stability after DNA damage. The mechanism by which this phosphorylation protects p27^Kip1^ against degradation is unclear. The ubiquitin-dependent proteolysis of p27^Kip1^ is regulated by distinct pathway, each operating during discrete phases of the cell cycle. One involves the ubiquitin ligase KPC, which targets p27^Kip1^ for degradation when it is exported to the cytoplasm in early G1 [27]. A second one operates in the late G1 through S and G2/M phases, and is dependent on the activity of the SCF^SKP2^ ubiquitin ligase, which recognizes phosphorylated p27^Kip1^ on T187 [28, 29]. Although the cytoplasmic export of p27^Kip1^ in early G1 is regulated by a nuclear export sequence (NES) located at the N-terminus of p27^Kip1^ (amino acids 32–45) [30], it is possible that phosphorylation at S140 interferes with p27^Kip1^ export by favoring novel protein interaction that would lead to p27^Kip1^ retention in the nucleus and protection from cytoplasmic degradation by KPC. Interestingly, the lysines ubiquitylated on p27^Kip1^ following phosphorylation at T187 have been mapped in the vicinity of the S140 phosphorylation site (K134, K153 and K165) [31]. Thus, if DNA damage occurs in late G1 cells, phosphorylation at S140 could contribute to protect p27^Kip1^ from degradation by interfering with the ability of the SCF^SKP2^ complex to recognize and ubiquitylate the lysines adjacent to S140 site and mark p27^Kip1^ for destruction. An alternative hypothesis is that phosphorylation at S140 could protect p27^Kip1^ against cleavage by proteolytic enzymes activated by DNA damage. Indeed, previous studies have demonstrated that p27^Kip1^ is cleaved on a caspase consensus site located just adjacent to S140 (D^136^PSD^139^S) after exposure to drugs inducing cellular and genotoxic stresses [32, 33]. Cleavage at this site generates a p23 fragment that favors a cytoplasmic localization at steady state due to the removal of the nuclear localization signal (NLS) located downstream (residue 152–167) [32]. Phosphorylation events within caspase consensus motifs have been found to either hinder or enhance caspase cleavage depending on the position of the phosphorylated residue and the nature of the substrate [34, 35]. In general, phosphorylation of the residue just downstream of the scissile aspartate has been shown to impair caspase proteolysis [34]. Thus, a possibility is that phosphorylation at S140 could impede its cleavage by activated caspases and favor the accumulation of p27^Kip1^ in the nucleus where it is needed to inhibit CDK activity. Although, the ectopically expressed p27S140A mutant localized to the nucleus (Fig 8C), it is possible that a caspase cleaved cytoplasmic fraction was beyond detection levels by immune-fluorescence or because of a decreased half-life.

Several studies have indicated that p27^Kip1^ plays an important role in the maintenance of genomic integrity. For example, mice with homozygous and heterozygous deletion of the p27^Kip1^ gene are both predisposed to tumor formation when exposed to gamma-irradiation or chemical carcinogens [16, 17]. In addition, in a mouse model of PDGF-induced oligodendrogliomas, p27^Kip1^ deficiency was shown to increase the occurrence of chromatid breaks and reduce the formation of Rad51 repair foci in response to IR [36]. More recently, a study using human primary and cancer cells, demonstrated that p27^Kip1^ protein stabilization was essential to maintain a late response cell cycle arrest after persistent exposure to genotoxic agents [18]. However, this late response was shown to be independent of ATM/ATR activation but rather downstream of p38 MAP kinase signaling. Our data demonstrating that phosphorylation of p27^Kip1^ at S140 occurs predominantly in the G0 and G1-phases of the cell cycle (Fig 7D) and that p27^Kip1^ is required to reduce CDK2 activity and prevent S-phase entry after DNA damage (Fig 2B and 2C) point toward a role for p27^Kip1^ in the G1/S DNA damage checkpoint. In this checkpoint, the cell cycle arrest generally occurs in a two-step response, referred to as initiation and maintenance [6]. The maintenance phase is a delayed response that is primarily dependent on ATM/ATR-mediated activation of p53 and its downstream target p21^Cip1^, which binds to and inhibits cyclin E/CDK2 complexes thereby arresting cells in G1 [37]. By contrast, the initiation phase is a transient and rapid p53-independent response that only lasts a few hours. In the latter, ATM-Chk2 and p38-dependent pathways rapidly, but transiently, prevent S-phase entry by promoting the degradation of cyclin D and the Cdc25A phosphatase that reverses the inhibitory phosphorylation on CDK2 [37]. The fast and transient pattern of S140 phosphorylation we observed suggests that p27^Kip1^ participates in the early initiation phase of G1/S checkpoint arrest (Fig 5). Our findings that the p27S140A mutant displays a marked half-life decrease in the 1 to 3 hour window post DNA damage (Fig 9E and 9F), which correspond to its peak of phosphorylation at S140 (Fig 5), suggests that rapid and transient stabilization of p27^Kip1^ also contributes to CDK2 inactivation, a required step to establish the G1 checkpoint arrest. The importance of the S140 phosphorylation is highlighted in our data obtained in breast tumorspheres showing that p27^Kip1^ is robustly phosphorylated at S140 and that the rescue-expression of the S140A mutant significantly impair the survival of these cells after induction of DNA damage (Fig 9D).

Although the p27^Kip1^ gene is rarely mutated in human cancers, its nuclear levels are often found decreased due to enhanced proteolytic degradation or cytoplasmic sequestration [15]. Our data demonstrating the importance of p27^Kip1^ in the establishment of a G1 checkpoint arrest downstream of ATM uncovers another layer to its tumor suppressing functions.

## Supporting Information

S1 Fig Densitometric quantification of CDK2 kinase activity from the experiment presented in Fig 2. MCF7 cells were transfected with non-targeting control or p27 siRNAs for 72h before treatment with 6 Gy of IR. Cells were harvested at the indicated time points post IR and CDK2 kinase activity was measured *in vitro* after immunoprecipitation using histone H1 as a substrate. CDK2 kinase activity level was normalized to total CDK2 levels detected by Western blotting. The data is presented as mean of 2 independent experiments ± SEM. Differences between groups were evaluated using two-tailed Student *t* tests among replicate experiments; *P < 0.05; **P < 0,005.
(TIF)

S2 Fig p27^Kip1^ is phosphorylated at S140 in different cell lines and localizes to the nucleus. (A) Western blot analysis of p27^Kip1^ S140 phosphorylation in different cell types. MDA-MB-231, U2OS and HFF (fibroblast) cells were treated with 0 (-) or 0.2 mM of H_2_O_2_ (+). Cells were harvested 1h later and analyzed by Western blotting with the indicated antibodies (left). (B) Immuno-localization of S140-phosphorylated p27^Kip1^. MCF7 cells were transfected with non-targeting control (-) or p27 siRNAs (+) for 72h and subjected to 0 or 6Gy of IR. 15 minutes post-irradiation, cells were fixed and analyzed by immunofluorescence microscopy with a p27 S140 phospho-specific antibody (p-p27(S140)) as indicated on the left of the panel. DAPI staining was used to mark the nucleus.
(TIF)

S3 Fig Densitometric quantification of p27^Kip1^ levels normalized to actin from the experiment presented in Fig 7C. MCF7 cells asynchronous (Async) or synchronized in G0, G1, G1/S or G2/M were analyzed by Western blotting for p27^Kip1^ levels 1h after treatment with 0 (-IR) or 6 Gy of IR (+IR). The data is presented as mean of 2 independent experiments ± SEM. Differences between groups were evaluated using two-tailed Student *t* tests among replicate experiments; *P < 0,0243. (B) DNA profiles of the synchronized cells from the experiment presented in Fig 7C. were obtained by flow cytometry analysis of PI incorporation. The percentage of cells present in each peak is indicated above the brackets.
(TIF)
