# Supplementary figures and images for: p27Kip1 Is Required to Mediate a G1 Cell Cycle Arrest Downstream of ATM following Genotoxic Stress
Source: PLoS One. 2016 Sep 9;11(9):e0162806. doi: 10.1371/journal.pone.0162806 (PMC5017644; doi:10.1371/journal.pone.0162806)

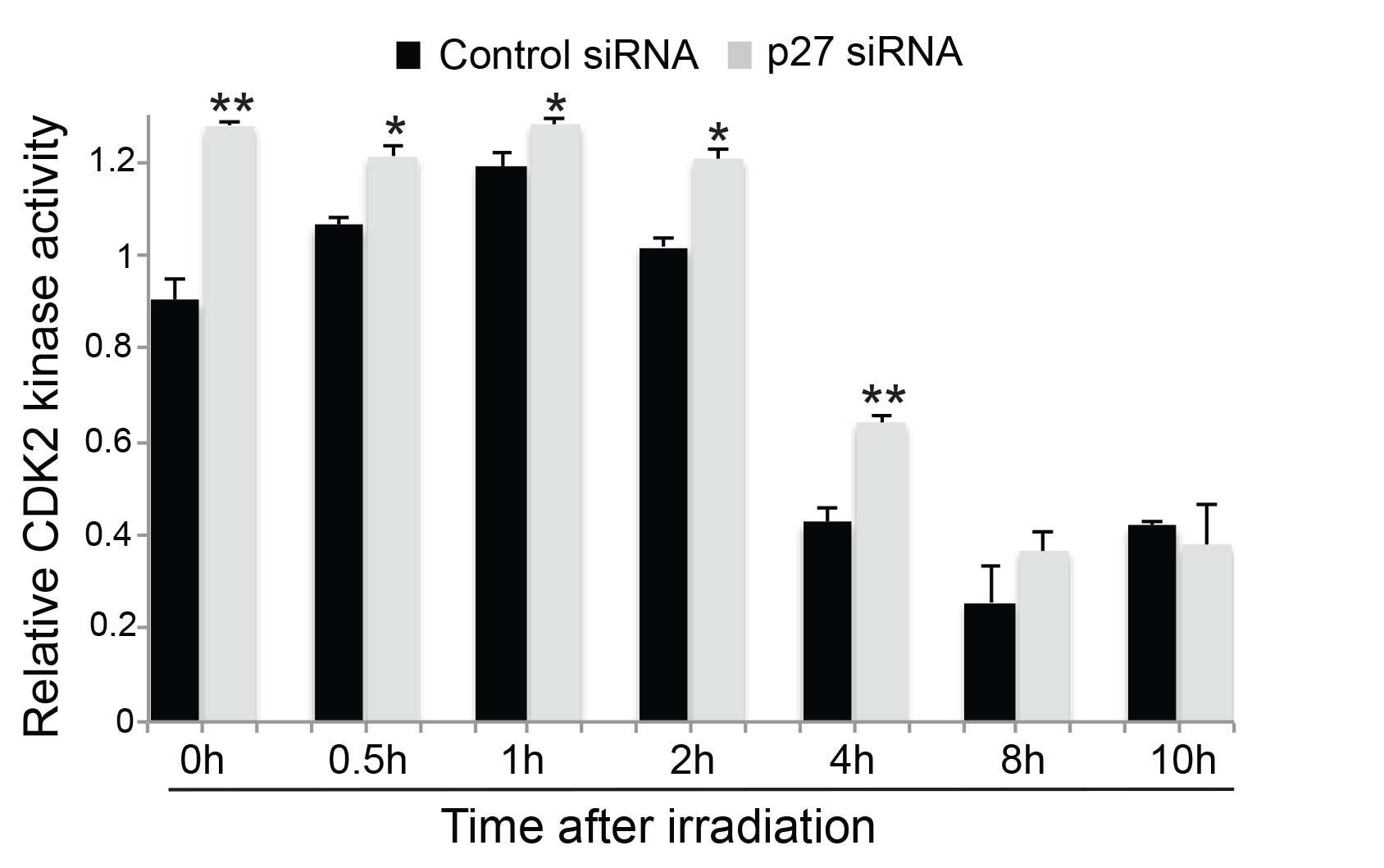

Supplement: S1 Fig — MCF7 cells were transfected with non-targeting control or p27 siRNAs for 72h before treatment with 6 Gy of IR. Cells were harvested at the indicated time points post IR and CDK2 kinase activity was measured in vitro after immunoprecipitation using histone H1 as a substrate. CDK2 kinase activity level was normalized to total CDK2 levels detected by Western blotting. The data is presented as mean of 2 independent experiments ± SEM. Differences between groups were evaluated using two-tailed Student t tests among replicate experiments; *P < 0.05; **P < 0,005. (TIF) [file pone.0162806.s001.tif]

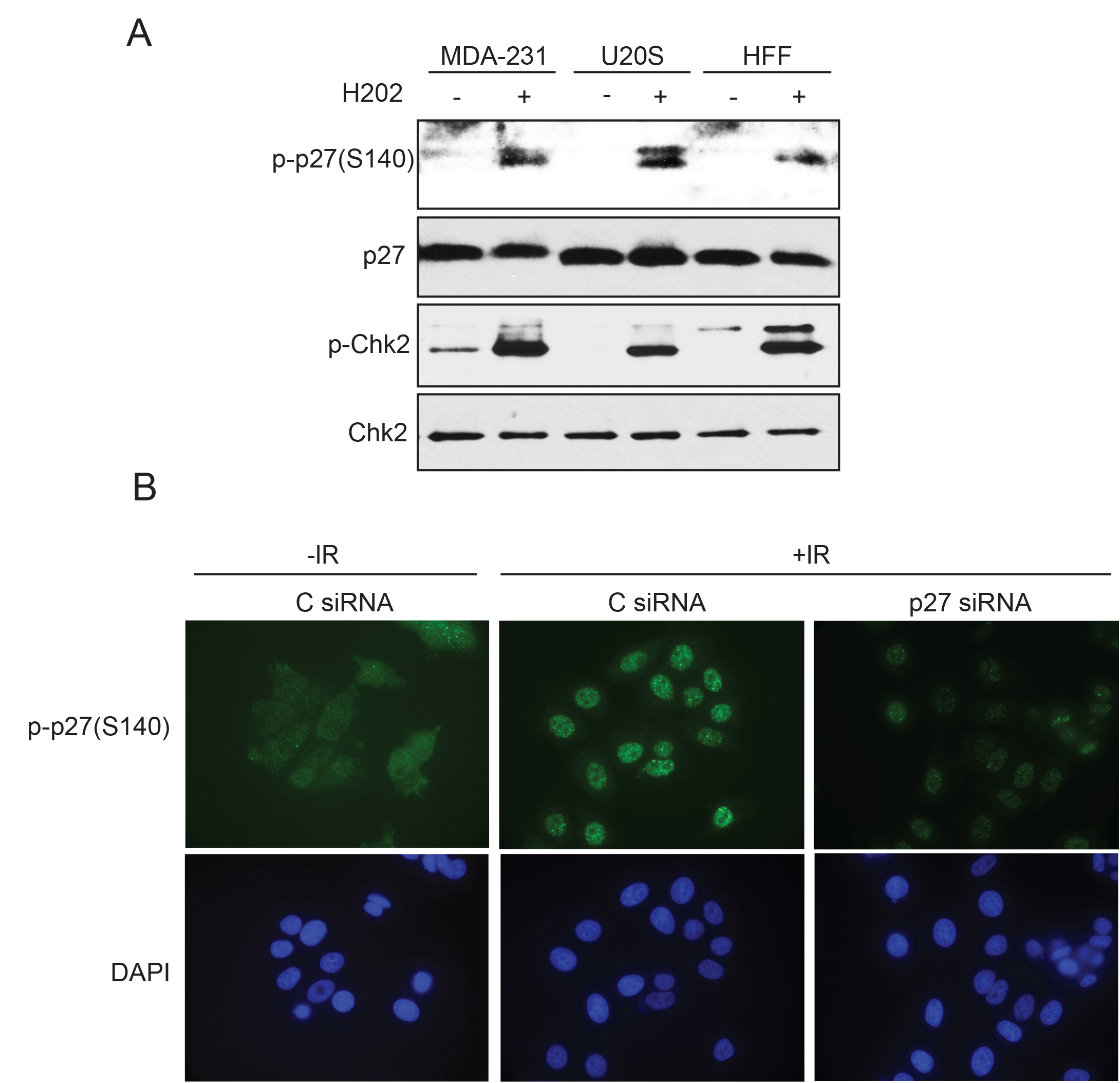

Supplement: S2 Fig — (A) Western blot analysis of p27Kip1 S140 phosphorylation in different cell types. MDA-MB-231, U2OS and HFF (fibroblast) cells were treated with 0 (-) or 0.2 mM of H2O2 (+). Cells were harvested 1h later and analyzed by Western blotting with the indicated antibodies (left). (B) Immuno-localization of S140-phosphorylated p27Kip1. MCF7 cells were transfected with non-targeting control (-) or p27 siRNAs (+) for 72h and subjected to 0 or 6Gy of IR. 15 minutes post-irradiation, cells were fixed and analyzed by immunofluorescence microscopy with a p27 S140 phospho-specific antibody (p-p27(S140)) as indicated on the left of the panel. DAPI staining was used to mark the nucleus. (TIF) [file pone.0162806.s002.tif]

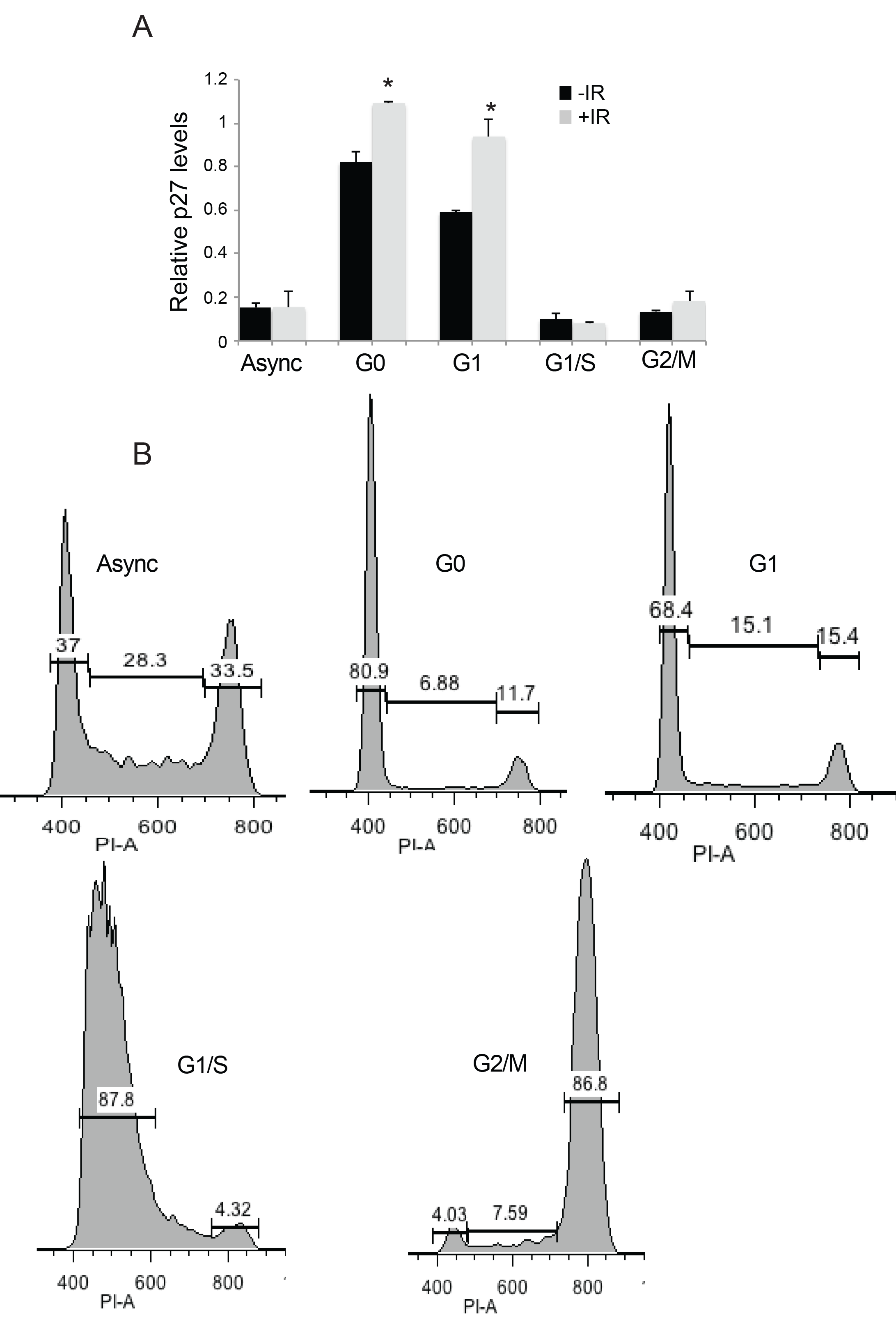

Supplement: S3 Fig — MCF7 cells asynchronous (Async) or synchronized in G0, G1, G1/S or G2/M were analyzed by Western blotting for p27Kip1 levels 1h after treatment with 0 (-IR) or 6 Gy of IR (+IR). The data is presented as mean of 2 independent experiments ± SEM. Differences between groups were evaluated using two-tailed Student t tests among replicate experiments; *P < 0,0243. (B) DNA profiles of the synchronized cells from the experiment presented in Fig 7C. were obtained by flow cytometry analysis of PI incorporation. The percentage of cells present in each peak is indicated above the brackets. (TIF) [file pone.0162806.s003.tif]
